# Measurement of hearing impairment among Greenlandic school-children: association between self-reported data and clinical examinations

**DOI:** 10.1186/s12887-022-03673-9

**Published:** 2022-10-26

**Authors:** Christina Schnohr, Jakob Schmidt Jensen, Cecilie Friis Skovsen, Preben Homøe, Birgit Niclasen, Ramon Gordon Jensen

**Affiliations:** 1 Section of Social Medicine, Department of Public Health, University of Copenhagen, Copenhagen, Denmark; 2 Department of Otorhinolaryngology, Head and Neck Surgery and Audiology, Rigshospitalet, University of Copenhagen, Copenhagen, Denmark; 3 Department of Otorhinolaryngology and Maxillofacial Surgery, Zealand University Hospital, Køge, Denmark; 4 Institute for Nursing & Health Science, Greenland Center for Health Research, University of Greenland, Nuuk, Greenland; 5 Allorfik, Niels Hammekensvej 41, P.O.Box 4323, 3900 Nuuk, Greenland

**Keywords:** Hearing impairment, Prevalence, Measurement properties, Methodology, School-children, Greenland

## Abstract

**Background:**

Multiple ear infections is causing hearing impairment among children all over the world and the health and social consequences track into early adolescence and later in life, if not treated. The monitoring of prevalence in a population is important to assess the need for interventions in a population.

**Methods:**

One hundred eighty  five children from 5 to 10^th^ grade from Sisimiut town and the nearby settlements participated in a clinical examination to have ear-examination and pure tone audiometry. Participants filled out a questionnaire at home with their parents before the clinical examination, and hearing impairment was collected as individual self-reports and as audiometric measurements.

**Results:**

A total of 185 children between 9 and 15 years of age (median: 11 years, IQR: 10–13) were included, 60% (*n* = 111) were girls. 247 (70%) of the 355 available otoscopies were clinically assessed as normal. Cohen’s Kappa coefficient was 0.31. Eighteen children (10%) were found to have hearing impairment. None of the children had hearing aids. Test performance for self-reports were that sensitivity was 56% and specificity was 87%. The predictive value of a positive test was 31%, and the predictive value of a negative test was 95%. 32 children (17%) reported hearing impairment to the extent that they were not able to keep up in school, of which half reported that it had lasted for more than one year. 7 of the 32 children reporting hearing impairment (22%) reported that the extent of their hearing impairment was affecting their classroom experience so they were not able to follow.

**Conclusion:**

Self-reported and clinically screening for hearing impairment are two different concepts. Even though the two concepts are statistically correlated, the correlation coefficients are low. The test performance indicated that self-reported data might be measuring hearing as an experience in a social environment and not directly comparable to pure tone audiometry which examines hearing in controlled testing conditions. Since both measure hearing impairment, they supplement each other in research on impaired hearing, and the choice of measure should relate to the purpose and method of the investigation.

## Background

Multiple ear-infections especially in early childhood is the leading cause of hearing impairment worldwide, and for main reasons such as poorer coverage of specialist doctors, and several risk factors present, the public health problem is especially profound in many indigenous populations [1, 2]. Several studies on the long-term consequences of an early onset of hearing impairment show evidence of compromised communication skills, poorer psychosocial behavior and emotional development, and it is shown to reduce academic performance [3, 4]. Most previous studies have been based on clinical examinations and only few studies are performed on representative samples of the target populations. Studies on self-reported hearing impairment are scarce.

The indigenous population in Greenland is known as a high risk population with regards to middle-ear infections [1], and concomitant conductive hearing loss. Mid-nineties studies of the young Greenlandic population found that 43% of children age 5 to 14 had hearing thresholds exceeding hearing loss [5]. More recent data has been collected in a study from 2010 where 223 Greenlandic children age 4 to 10 years old was examined, and found that 20% had middle-ear infections at some point in childhood whereof 91% had developed some degree of permanent hearing loss [6, 7]. Jensen and colleagues studied 438 individuals from age 11 to 24, and found hearing loss between 2 and 50% depending on the use of either the American Speech-Language-Hearing Association (ASHA) or World Health Organization (WHO) definition at that time (2011) [7]. According to the former WHO criteria a person had a hearing loss if the pure tone average (PTA) was worse than 25 decibel (dB) in the better ear. The current WHO definition states that a person with a PTA worse than 20 dB in any ear has a hearing loss [8].

A recent study examining 185 children age 9 to 15, and found that 29% had hearing impairment using PTA of 15 dB for either low or high frequencies or both as threshold, and 10% had hearing impairment when using 25 dB as threshold [9]. Questionnaire-based data have been collected from Health Behavior in School-aged Children (HBSC) Greenland, which is a national cross-sectional survey among all school-children in Greenland from class 6 to 10, collected self-reported data on hearing impairment, and from data on 2.273 children age to 10 to 18, 14% responded ‘yes’ to the question ‘Does poor hearing prevent you from keeping up in class?”^1^ [10].

The research area of hearing disabilities in the Arctic region has historically received a lot of attention from both ear-nose-and-throat (ENT) doctors and epidemiologists. Whereas ENT doctors has a focus on the pathophysiology of ear infection, treatment modalities and complications such as hearing loss, at the individual level, large epidemiological surveys aim to study public health impacts, social, psychological and emotional consequences of hearing impairment. Public health advocates and policy makers are interested in monitoring the prevalence of hearing loss in the population to support the individual for the benefit of the society, and to prevent public health at a broader level from worsening.

Reports of 10–20% of children having hearing impairment means that many individuals are suffering from compromised communication skills and worsening of psychosocial and emotional development (3;4). At a societal level, reported consequences has been of poorer reading skills, behavioral problems and lower IQ can affect the healthy development of future generations [4, 11–14].

Pure tone audiometry is considered the gold standard for measuring hearing loss in population-based studies and screenings, but clinical measures vary according to definitions of threshold levels. Furthermore, audiometric outcomes can be limited by ambient noise, examiner competence and the developmental stage and behavior of the child.

When using self-reported measures on hearing loss collected from questionnaire data, the validity of the questions are depended on the child’s motivation and interest in the questionnaire and the developmental stage and behavior of the child. A lack of self-report measures to use for children and adolescents – who may never have experienced normal hearing, and would therefore not be able to identify a change in hearing levels (from normal to impaired) – led to the development of items for a questionnaire-based survey among Greenlandic school-children [10]. In 2017 to 2018 items were developed to include in the national questionnaire-based survey on adolescent health, HBSC Greenland. The study by Schnohr et al. describe the process of developing questions for epidemiological surveys to assess impaired hearing in a national sample [10], but no previous studies have compared the self-reported data with data collected in a clinical setting with pure tone audiometry. Therefore, the present paper examine the association between the items developed for the questionnaire-based survey and clinical measures of hearing impairment considered as a golden standard.

## Methods

This present cross-sectional study included school-aged children in Sisimiut in Greenland. All children from 5 to 10^th^ grade from the two schools in Sisimiut and the nearby settlements were invited to participate, corresponding to 422 children in total. Data was collected in September 2020.

Participants filled out a questionnaire with their parents or guardians before ear-examination and audiometry, and parent or guardian provided informed consent before taking part in the clinical examinations.

### Questionnaire

The questionnaire used was made from a base of the questions from the Greenlandic contribution to the international Health Behaviour in School-aged Children (HBSC) study. The questionnaire focused on the self-reported questions about hearing impairment, and also included questions on social factors relevant to hearing impairment and risk factors for the development of hearing related diseases. The questionnaire was available in Danish and Greenlandic.

As described by Schnohr et al. (2019) the item measuring self-reported hearing impairment was formulated;*”Does poor hearing prevent you from keeping up in class?”* with the response categories; *Yes* and *no* [10]. If the respondent replied positively, the questionnaire would guide the respondent to three additional questions, with the following supplementary text; “*You answered YES to poor hearing preventing you from keeping up in class*:” and asking respondents to reply to the following question on extent of hearing impairment;*”How much does poor hearing prevent you from keeping up in class?”* with the response categories; *A little*, *Quite a lot*, and *I can’t keep up at all*., a question on the source of knowledge to the hearing impairment;*”How do you know you have poor hearing? (multiple answers allowed)”* with five response categories; *I noticed myself, Someone in my family told me, One of my teachers told me, One of my friends told me* and *A doctor/nurse/health visitor told me*., and lastly a question on the duration of impaired hearing;*”For how long have you suffered from poor hearing?* with five response categories; *Less than 3 months*, *More than 3 months but less than a year, 1–5 years, 5–10 year,* and *More than 10 years*.

As described by Schnohr et al. [10], the face and content validity of the questionnaire items, had been tested in cognitive interviews with the target group and nine bilingual Greenlandic school-children, who had been recruited in Nuuk and Ilulissat. After completion of a short questionnaire containing only the items listed above, the school-children were interviewed on the verbal probing about the participants’ general impression, understanding and what they were thinking about when responding to the questions. All findings were discussed between bilingual health researchers and psychometric experts, and edits were made to shorten the questions and adaptations were made on the wording, so the reading requirements of the respondents were as low as possible for optimal validity.

### Clinical examinations

The clinical examinations are described in our earlier work by Jensen et al. (2021). All examinations were conducted in a room in the schools, selected to reduce background noise. All respondents were examined for 15–30 min including a conversation about the questionnaire and an audiological examination. The audiological examination was conducted by a medical student (JSJ) who prior to data collection had received simulator based training and clinical teaching at the Department of Otorhinolaryngology, Head and Neck Surgery and Audiology, Rigshospitalet, Denmark. All examinations and conversations with respondents were followed by a Greenlandic research assistant who supported all work during the examinations, including connecting with school-children as respondents and before, during and after the inclusion into the study and the examination.

For the audiometry an Interacoustics Callisto™ audiometer was used with a TDH39 headset. Air conduction (AC) thresholds were obtained at six frequencies: 250, 500, 1000, 2000, 4000, and 6000 Hertz (Hz). Thresholds were determined manually according to guidelines from the American Speech-Language-Hearing (ASHA) Association. Hearing impairment was categorized into *with hearing impairment* and *without hearing impairment.* The definition of hearing impairment was based on the PTA for the low frequencies (i.e., 500, 1000, and 2000 Hz) and for the high frequencies (i.e. 4000 and 6000 Hz. A child was considered to have a hearing impairment if the PTA for either the low or high frequencies were > 25 dB in any ear [9].

### Statistical analyses

Analyses was performed to assess the statistical association between self-reported (SR) and clinically measured (CM) hearing impairment.

A simple 2 × 2 table initially described the association between the SR and CM, and false positives as well as false negatives were examined. Secondly Cohen’s Kappa coefficient was calculated to assess the agreement between the two categorical items. The underlying assumption of using the Kappa coefficient is that the two categorical items tested are measuring the same construct. Univariate logistic regression examined SR by CM to assess the statistical association. The test performance was examined by calculating sensitivity, specificity and positive and negative predictive values.

## Results

A total of 185 (44%) of the invited children between 9 and 15 years of age (median: 11 years) participated whereof 111 (60%) were girls. Thirty-two of the 185 respondents answered ‘yes’ to the question whether they were not able to follow class because of poor hearing, corresponding to 17%. When studying Table 1 further, it is worth highlighting that out of the 18 with clinically measured impaired hearing, almost half of them did not report a problem, and had not themselves recognized having a problem following school. Out of the 167 with normal hearing according to the clinical measures, 13% reported not to be able to follow school due to hearing problems. If reading the Table vertically, 22 out of the 32 students reporting impaired hearing as a cause of not being able to follow school was not classified as having a clinically noteworthy impaired hearing. Additionally, out of the 153, who themselves reported normal hearing, 5% had impaired hearing from the audiological examinations. Cohen’s Kappa coefficient was 0.31 which indicates a very low agreement (Table 1).

**Table 1:** Self-reported hearing impairment by clinically measured hearing impairment

		Clinically measured		
		Normal	Impaired	TOTAL
Self-reported	Normal	145 (true negative TN)	8 (false negative FN)	153
	Impaired	22 (false positive FP)	10 (true positive TP)	32
	TOTAL	167	18	185
Simple Kappa Coefficient			95% LCL	95% UCL
	Kappa	0,3146	0,1314	0,4979

Univariate logistic regression showed an odds ratio (OR) of 8.2 (95% CI 2.9–23.1) of SR when having been classified with hearing impairment by CM.

Further examination was done by calculating the $sensitivity=\frac{\mathrm{TP}}{\mathrm{TP}+\mathrm{FN}}$, 10/18 = 56%, the $specificity=\frac{\mathrm{TN}}{\mathrm{FP}+\mathrm{TN}},$ 145/167 = 87%. Predictive value of a positive test $PPT=\frac{\mathrm{TP}}{\mathrm{TP}+\mathrm{FP}}$ was 10/32 = 31%, and predictive value of a negative test $PNT=\frac{\mathrm{TN}}{\mathrm{FN}+\mathrm{TN}}$ was 145/153 = 95%.

## Discussion

When basing conclusions on impaired hearing among school-children in Sisimiut, the prevalence varies from 17% according to self-reports, to 10% according to clinical examination from the threshold chosen in the present survey. Choice of threshold levels determines the variation between the two types of measurement, but there are large inconsistencies identified when associating the two measures.

Sensitivity measures the test’s ability to classify the ones with hearing loss correctly, and the result of 56% shows poor ability to do so. Specificity measures the test’s ability to classify the students with normal hearing correctly, and this happened in 87% of the measures.

The likelihood of actually having impaired hearing when reporting a positive test result by self-reports were only 31%, which shows that other factors are at play when children are responding to a question on their hearing. The likelihood of having normal hearing when reporting to have normal hearing in the questionnaire were as good as 95%. Given that the sensitivity and positive predictive values are relatively low indicate that the self-reported measures should be seen as an indication of problems, that could benefit from being examined further.

Testing with Kappa coefficient was done to include a traditional measure of correlation, which underlines that the two measures are not closely related. Kappa coefficient is used to measure inter-rater reliability, and taking account the possibility of an agreement occurring by chance. Since the assumption of using Kappa is that the two measures are related to the same underlying construct seems to be rejected.

With these findings, it becomes apparent, that researchers and clinicians should allow for a “grey zone” of classifications including the personal assessment of hearing impairment. This “grey zone” can be quantified by further analyses of the 30 out of 185 children that showed inconsistencies between the SR and CM, that were first evaluated for a second opinion, and initial findings were confirmed. Given the degree of variations, a valid conclusion is to consider the two measurements of hearing impairment as covering different areas, and one explanation is that one is a consequence of the other. This is supported by a strong statistical association of SR by CM, with an OR of 8.2.

As described by Schnohr et al. (2019) the item measuring self-reported hearing impairment was associated to the school-situation, since the item was developed for school-aged children. In the event, that a respondent was asked why he/she did not keep up in school, and the suggestion was made that is was due to impaired hearing. Such a question can simply nudge the respondent to reply ‘yes’ even though the underlying cause – to not paying attention to school – is something else. Often children are asked to pay attention for other reasons than impaired hearing, and it would be relevant to adjust for those related causes. Age, gender, socioeconomic background and overall self-perceived health has been demonstrated to be associated with disagreement between self-reported hearing and audiometric outcome among older adults and may also affect the responses of children [15, 16]. However, in a sample size of 185 respondents, it is not statistically possible to examine associations of related predictors on impaired hearing, to e.g. study the school environment and other aspects of the respondent’s health. This would be interesting and relevant for future studies.

Important factors that could lead to self-reported hearing difficulties in school but a normal pure-tone audiogram at examination are auditory neuropathy spectrum disorders, auditory processing disorders and any attention deficit disorder. Furthermore, the acoustic environment in the school is important. Background noise in the classroom, size of the room, number and position of students will affect their ability to hear the teacher’s voice.

In Greenland it is only possible – as in this study – to use pure tone audiometry. Hearing is a highly complex neurological process that involve not only the outer and inner ear but also lower and higher cerebral functions that are interacting and are influenced by psychological processes and by the broader context in which the hearing takes place. The individual perception of what is heard therefore is a meta process.

Considerations can be taken to what the measurement properties are for each of the two methods, and taking into account if the conclusions derived should stem from a representative result for the entire population – in which case an epidemiological survey of a large representative samples of the population is most appropriate – or whether researchers are looking for clinical conditions causing hearing impairment – in which case clinical examinations of high risk populations is most appropriate.

Among older populations audiometric measures was found to explain less than 50% of the variance in hearing handicap and it was suggested that hearing handicap in the elderly will be measured more appropriate via a self-report format rather than as an inference from audiometric data [17].

In spite of findings that the two measurements are not measuring the same construct, it is clear that they are closely related. Irrespectively where the threshold levels are set, it is fair to presume that students that are classified as normal hearing will have problems following school, and it becomes evident that both measures have each their relevance, depending on the purpose and method of any given investigation. Based on the present study, it is not appropriate to conclude whether the self-reported or the measured collected from clinical examinations generally have the highest reliability, as both measurements have advantages and uncertainties, that could benefit from being taken into account.

The Arctic population is a high-risk population for chronic conditions related to impaired hearing, and a continued focus on the monitoring and examinations of both representative and high-risk populations serves as an important topic for future public health in Greenland. The authors of the present paper are continuing to develop questionnaires to improve the monitoring of impaired hearing in Greenland, and the potential of e.g. using mobile phones for testing to improve the reach of clinical staff is one of the areas worth examining further.

## Conclusions

The two measurements of hearing impairment compared in the present study seems to cover substantially different areas, since there are large inconsistencies identified when correlating the two measures. Since both measure hearing impairment, they supplement each other in ways not yet examined into details. Results of a clinical test of hearing is challenged if the test conditions are not optimal. An optimal test condition of quiet space and controlled environment never reflects to everyday situations where the level and quality of hearing is an assessment perceived by an individual from a given situation. A number of conditions are relevant to consider when measuring a person’s hearing, and in the case of children in a school-setting, the perception of hearing may vary according to communication skills, problems with concentration, school readiness or even behavioral problems often referred to as causes of “not paying attention” or not being able to listen. The reliability of the different measures of hearing is relevant to examine further in studies, where the controlled clinical examinations is performed in connection to interviews on situations, where the hearing in a social environment can also be assessed.

## Data Availability

The dataset used and analysed during the current study are available from the corresponding author on request.

## Abbreviations

ASHA: American Speech-language Hearing Association
WHO: World Health Organization
PTA: Pure Tone Audiometry
dB: Decibel
HBSC: Health Behavior in School-aged Children
ENT: Ear-nose-throat
AC: Air Conduction
SR: Self-reported
CM: Clinically measured
OR: Odds ratio
LCL: Lower confidence limit
UCL: Upper confidence limit

## Footnotes

1 Greenlandic version:’ Tusaasaqarniarnerit atuarninni malinnaaninnut ajoqusiiva?’, Danish version: ‘Forhindrer dårlig hørelse dig i at følge med i skolen?’.
